# Preparation of Bio-Based Polyamide Elastomer by Using Green Plasticizers

**DOI:** 10.3390/polym8070257

**Published:** 2016-07-14

**Authors:** Miaomiao He, Zhao Wang, Runguo Wang, Liqun Zhang, Qingxiu Jia

**Affiliations:** 1 Key Laboratory of Beijing City on Preparation and Processing of Novel Polymer Materials, Beijing University of Chemical Technology, Beijing 100029, China; yflhmm@126.com (M.H.); wangrunguo@126.com (R.W.); 2 Department of Chemistry, South Dakota School of Mines and Technology, Rapid, SD 57701, USA; wangzhaoqd@163.com; 3 College of Materials Science and Engineering, Beijing Institute of Fashion Technology, Beijing 100029, China

**Keywords:** itaconic acid, bio-based, amorphous polyamide, plasticization, elastomer

## Abstract

The purpose of this work was to study the effects of three green plasticizers H_2_O, glycerol, and soybean oil, on the properties of bio-based BDIS polyamides. The BDIS polyamides synthesized from the following biomass monomers: 1,4-butanediamine (BD), 1,10-decanediamine (DD), itaconic acid (IA), and sebacic acid (SA). It is interesting to note that the amorphous BDIS (IA-80%) polyamide was changed from the glassy state to the rubbery state after water soaking and induced crystallization at the same time. The H_2_O-plasticized non-crosslinked BDIS (IA-80%) polyamides can be very useful for the preparation of physical water gel. The glycerol- and soybean oil-plasticized BDIS (IA-80%) polyamides displayed excellent toughness. The plasticized BDIS (IA-80%) polyamides were characterized by Fouriertransform infrared spectroscopy (FTIR), differential scanning calorimetry (DSC), thermogravimetric analysis (TGA), mechanical testing, and X-ray diffraction (XRD).

## 1. Introduction

Polyamides (PAs), with their versatile properties and wide applications, play an essential role in our daily life. In particular, PA 6 and PA 66 are well known as materials with relatively high strength, high ductility, high abrasion resistance, excellent resistance to short-term heat exposure and good chemical resistance [1,2,3,4]. However, polyamides are known to be hygroscopic, because of the existence of a large number of hydrophilic amide groups, which can hydrogen bond with the sorbed water molecules. Therefore, water is an important plasticizer for polyamides.

Plasticization with water lowers the glass transition temperature of the polyamides which, in turn, reduces the stiffness of the material and enhances the toughness [5]. It is generally accepted that water is absorbed in the amorphous phase that induces reorganization of the hydrogen bond structure [6,7]. However, the amorphous phase is not homogeneous, but consists of regions with different mobility [8,9]. Litvinov et al. indicated that, in an amorphous phase of polyamide 6, the water mainly resides in the softest part [10]. Furthermore, it was recently shown that solvation of amide groups by water molecules, inhibiting crystallization, and/or allowing the dissolution of crystallites, as observed in water-immersed Nylon 46 [11,12,13]. Amide group interactions may be efficiently shielded by aqueous ionic solutions as well [14].

**Scheme 1:**
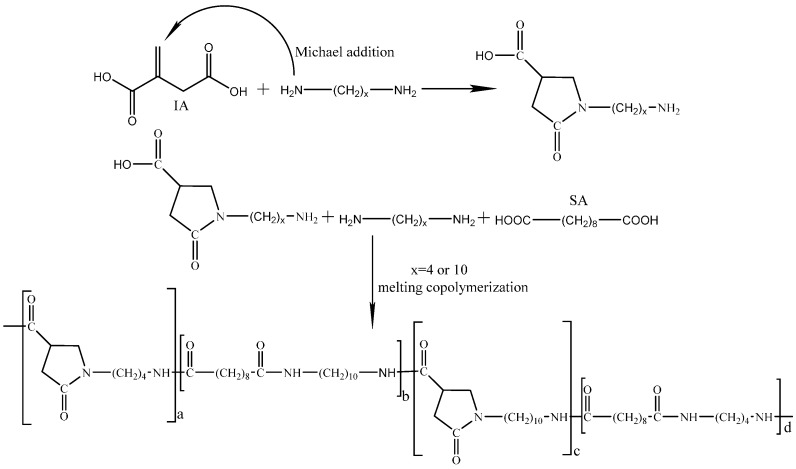
Structure of BDIS (IA-80%) polyamides (a, b, c, and d are integers).

To meet the growing demand for sustainable development, we need to explore renewable raw materials to synthesize bio-based and high-performance polyamides. In our previous work we reported the synthesis, and the structural and thermal characterization of fully renewable BDIS [15] copolyamides from itaconic acid (IA), sebacic acid (SA), 1,10-decanediamine (DD) [16], and 1,4-butanediamine (BD) [17]. At the IA content of 80%, the transparent amorphous BDIS polyamide exhibit excellent mechanical properties, including high modulus, yield stress, tensile strength, and an elongation at break of over 300%. Interestingly enough, the fully amorphous BDIS (IA-80%) polyamide became an elastomer after being plasticized with water. Additionally, the amorphous BDIS (IA-80%) polyamide was induced crystallization after water soaking. IA was replaced by SA and reacted with diamine to generate the pyrrolidone ring [18]. The structure of the amorphous BDIS (IA-80%) polyamide is shown in Scheme 1. The pyrrolidone ring could increase the molecular spacing and the chain irregularity, which affects the properties of the amorphous BDIS (IA-80%) polyamides. As of now, there are few commercial products of amorphous polyamides or rubbery state polyamides.

In addition, as the plastic industry continually develops, the search for bio-based plasticizers is important in the development of new bio-based polymers from renewable resources. Glycerol, as a hydrophilic plasticizer, is generally used for protein-based polymers, such as to improve mechanical properties [19,20,21]. Soybean oil, as a green hydrophobic plasticizer, is chosen as a contrast sample compared with the hydrophilic plasticizers.

Therefore, the purpose of this investigation was to study the plasticizing efficiency of water, glycerol, and soybean oil on the microstructure, crystallization, and mechanical properties of fully-amorphous BDIS (IA-80%) polyamides. The plasticizing effect was investigated by using various experimental techniques, like FTIR, DSC, XRD, and mechanical tests. These plasticized amorphous BDIS (IA-80%) polyamides, which were obtained from biomass monomers, and water-plasticized BDIS (IA-80%) polyamide showed good bio-compatibility (as shown in supplementary information Figure S1), which makes it useful for applications in biomedical materials (carrier materials of externally-applied medicine and even tissue engineering materials). Although, the water-plasticized BDIS (IA-80%) polyamides are linear materials, and the water molecules will evaporate; but they could still be used in applications in an aqueous environment which does not need to consider the evaporation of water, such as contact lenses or water filtration membrane material. Therefore, it is reasonable to assume that the plasticized BDIS (IA-80%) material could find wide applications.

## 2. Materials and Methods

### 2.1. Materials

Itaconic acid (≥99%), sebacic acid (≥99%), and 1,4-diaminobutane (≥99%) were purchased from Alfa-Aesar Chemical Inc. (Ward Hill, MA, USA). 1,10-diaminodecane (≥98%) and glycerol (≥99%) were purchased from HWRK Chemical Inc. (Beijing, China). The 1,10-diaminodecane was purified by recrystallization three times. Soybean oil, a gift from Guangzhou Dongfeng Chemical Industrial Co., Ltd. (Guangzhou, China), was dried in a vacuum oven at 80 °C for 6 h. Hydroquinone (99%) and orthophosphoric acid (99%) were purchased from Sinopharm Chemical Reagent Beijing Co., Ltd. (Beijing, China).

### 2.2. Synthesis of Bio-Based Polyamides

The preparation of bio-based aliphatic BDIS polyamides was described in previous studies [15,22]. The BDIS polyamides were synthesized through a two-step process: the formation of ammonium salts and subsequent melting-polycondensation. Equimolar amounts of diacids (IA and SA) and diamines (DD and BD) were dissolved in ethanol, and the mixture was stirred slowly for 30 min at 60 °C to allow the formation of the ammonium salt. The salt was obtained as fine white powder after elimination of the solvent and dried in a vacuum oven at 40 °C for 12 h before use. The BDIS polyamides were synthesized by allowing the salts to react with each other. Reagents were charged in a three-necked flask equipped with anitrogen inlet, a central mechanical stirrer, a distillation head connected to a condenser, and a receiver flask. The inhibitors hydroquinone and orthophosphoric acid, at 0.05 wt % and 0.01 wt % of the total mass of monomers, respectively, were also added into the flask. The flask was placed in an oil bath at 180 °C for 1 h. The temperature was increased first to 200 °C and kept constant for 2 h. It was then further increased to the final temperature of 210 °C and kept constant for another 1 h, with a pressure reduction to 300 Pa. The BDIS (IA-80%) polyamide was prepared with the same monomer compositions. The molar ratio of BD to DD was fixed at 1:1, and the mole ratio of IA to SA was fixed at 4:1. The BDIS (IA-80%) polyamide was yellowish and transparent at ambient temperature and had a viscosity-average molecular weight of 56,725 g/mol and a glass transition temperature of 58 °C [23]. The BDIS (IA-80%) polyamides had excellent mechanical properties, including high modulus, tensile strength, yield stress, and elongation at break (over 300%).

### 2.3. Plasticizing Experiments

The bar specimens (1.0 mm thick) were prepared by vacuum pressing and soaked in a plasticizer (deionized water, glycerol, or soybean oil) at room temperature (23.0 ± 0.1 °C). The percentage swelling of a specimen was measured according to Chinese Standard GB/T 1034-2008. The specimen was taken out of the plasticizer at various times and its weight measured with an analytical balance immediately after its surface was wiped dry with a filter paper. The plasticizer content (PC) of the specimen was calculated as follows:

(1) $$\mathrm{PC}(\%)=\frac{W_{1}-W_{0}}{W_{0}}100$$

where *W*_0_ is the weight of the dry specimen and *W*_1_ is the weight of the plasticized specimen. Each measurement was carried out in quintuplicate, and the standard deviation calculated.

### 2.4. Determination of Hydrogen Bonding by Molecular Dynamics Simulation (MD)

**Figure 1:**
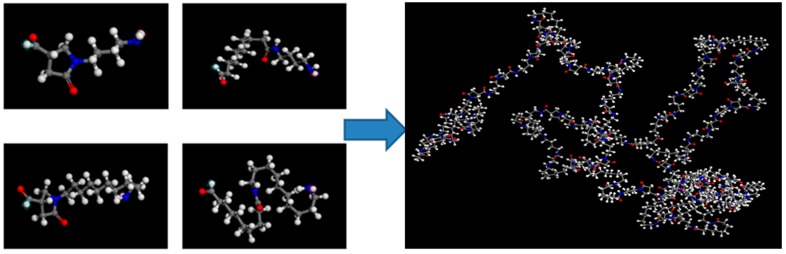
Models for MD simulation of BDIS (IA-80%) polyamides (in the unit cell: red, O; white, H; gray, C; blue, N).

**Figure 2:**
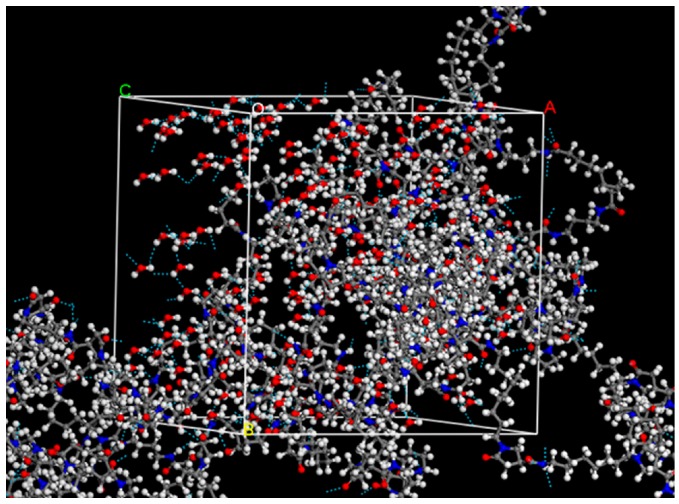
MD simulation of hydrogen bonds (blue dashed lines) by using Materials Studio software.

For the molecular dynamics simulations, the Discover and Amorphous Cell module of the Materials Studio software (Accelrys, San Diego, CA, USA) suite was used [24]. All of the calculations were performed by using the condensed-phase optimized molecular potentials for atomistic simulation studies (COMPASS) force field. The COMPASS force field has been widely used to predict the structures and properties of polymers. The initial velocities were assigned by using the Maxwell-Boltzmann profiles at 298 K. The Verlet velocity time integration method [25] with a time step of 1 fs was used for calculations. Figure 1 shows that a BDIS (IA-80%) polyamide is constructed by four repeating units, including two pyrrolidone ring structures and two linear structures. Amorphous cells were constructed of blends of two chains of 50 repeat units of BDIS (IA-80%) polyamide. First, the BDIS (IA-80%) polyamide chains and plasticizers were built in a periodic boundary cell (Figure 2). Then, the cell was taken by energy minimization to the lowest energy. The systems were equilibrated in the isothermal-isobaric ensemble at 298 K. Finally, the cell was used to analyze hydrogen bonds.

### 2.5. Characterizations

#### 2.5.1. Fourier Transform Infrared Spectroscopy (FTIR)

The adsorption spectra of plasticized BDIS (IA-80%) polyamide samples were recorded on a TENSOR 27 Fourier transform infrared spectrophotometer (Bruker, Rheinstetten, Germany) equipped with a Smart Orbit diamond-attenuated total reflection (ATR) accessory. The spectra were collected in the wavenumber range 500–4000 cm^−1^ at a resolution of 4 cm^−1^, and the signal averaged over 32 scans. FTIR spectra of water-plasticized BDIS (IA-80%) polyamide were baseline-corrected and normalized to the average of the C=O peaks at 1643 cm^−1^. FTIR spectra of glycerol and soybean oil were baseline-corrected and normalized to the average of the N–H bending(amide II band) at 1540 cm^−1^.

#### 2.5.2. Differential Scanning Calorimetry (DSC)

DSC measurements of plasticized BDIS (IA-80%) polyamide samples were performed on a DSC1 STARe system (Mettler-Toledo International Inc., Greifensee, Switzerland) equipped with a liquid nitrogen cooling accessory. Typical total sample weights of about 5mg were used. The DSC runs were performed using aluminum crucibles. Depending on the experimental conditions desired, the crucible was used a pierced lid (thus limiting the surface available for mass transfer to a 1 mm diameter hole). The sample was heated from room temperature to 220 °C at a heating rate of 10 °C/min. Data were recorded by scanning in the temperature range from room temperature to 220 °C at a heating rate of 10 °C/min. *T*_g_ was determined from the mid-point of the observed change in heat capacity.

#### 2.5.3. Thermogravimetric Analysis (TGA)

In order to determine the plasticizer loss in the BDIS (IA-80%) polyamides, we conducted thermogravimetric analysis on a STARe system TGA/DSC1 thermogravimeter (Mettler-Toledo International Inc., Greifensee, Switzerland) equipped with a cooling water circulator. A sample of approximately 10 mg was heated at a scanning rate of 10 °C/min from room temperature to 650 °C, with a nitrogen purge of 20 mL/min, and the loss of weight was recorded.

#### 2.5.4. X-Ray Diffraction (XRD)

XRD measurements were carried out on a Rigaku RINT diffractometer (Tokyo, Japan) with a copper Kα radiation (λ = 1.54 A°, 40 kV, and 200 mA) in the 2θ range of 5° to 65° at a scan rate of 5 °/min.

#### 2.5.5. Polarized Optical Microscopy (POM)

POM observations were performed under a BX90 microscope (Olympus Co., Tokyo, Japan) equipped with a digital camera. A BDIS (IA-80%) polyamide thin film (0.1 ± 0.01 mm) was sandwiched between two glass slides and soaked in water, glycerol, or soybean oil. The images were recorded after the completion of crystallization.

#### 2.5.6. Mechanical Properties

The tensile strength, elongation at break, and Young’smodulus of the plasticized BDIS (IA-80%) polyamide samples were determined at 23 ± 1 °C on a CMT 4104 testing machine (Shenzhen SANS Testing Machine Co., Ltd., Shenzhen, China) equipped with a 50-N load cell. The dumbbell specimens (65 mm ×3.14 mm × 1mm) were prepared by vacuum pressing and tested at a crosshead speed of 10 mm/min until breakage. For each plasticized BDIS (IA-80%) polyamide sample, the test was performed five times, the results were averaged, and a standard deviation obtained. In addition, the BDIS (IA-80%) polyamide samples after hydration were subjected to cyclic loading-unloading experiments.

## 3. Results and Discussion

**Figure 3:**
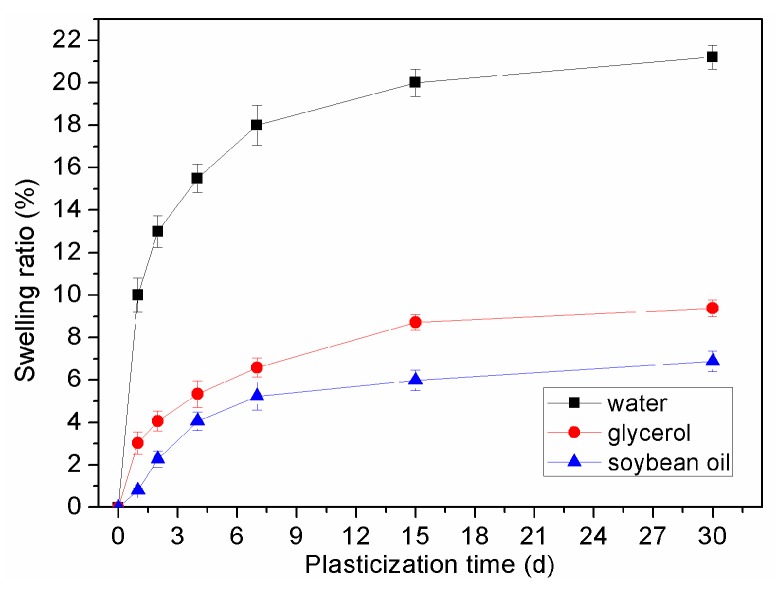
Swelling ratios of BDIS (IA-80%) polyamide at different plasticization times.

Plasticizer content is an important estimate of the plasticizer efficiency. With increasing the contents of the three plasticizers, the swelling ratio of BDIS (IA-80%) polyamide increases. The swelling ratios of BDIS (IA-80%) polyamide related to the plasticization time is shown in Figure 3. After plasticization for 30 days, the absorption of three plasticizers has almost reached equilibrium. At the same plasticization time, the swelling ratio of BDIS (IA-80%) polyamide plasticized with soybean oil is lower than that of the BDIS (IA-80%) polyamide plasticized with water or glycerol. The swelling ratio of the BDIS (IA-80%) polyamide plasticized with water is the highest of the three plasticized BDIS (IA-80%) polyamides. The BDIS (IA-80%) polyamide has a water uptake of almost 25.0% after being soaked in water for 30 days. This water uptakeis higher than those of traditional polyamides, such as PA 6 [26] and PA 66 [27]. Since the BDIS (IA-80%) polyamide is totally amorphous, the interactions of the BDIS (IA-80%) polyamide macromolecular chains are significantly weaker than those of traditional polyamides, which is useful for the preparation of the physical water gel.

### 3.1. Chemical Structure by FTIR

**Figure 4:**
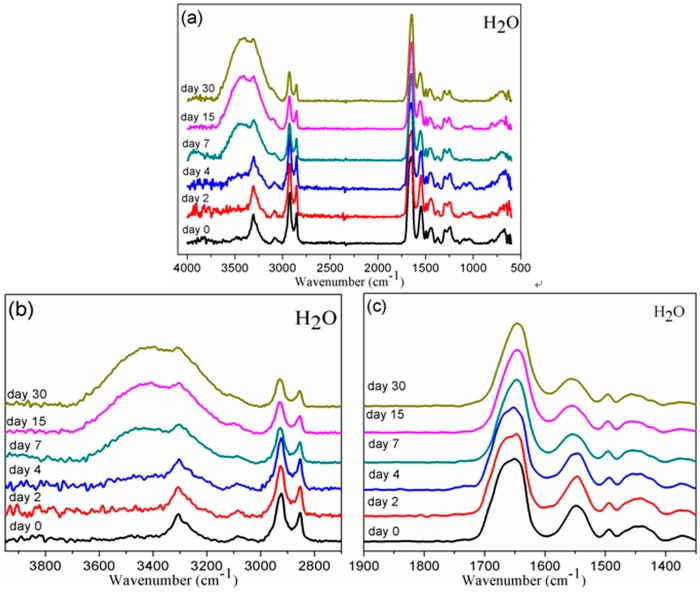
FT-IR spectra of BDIS (IA-80%) polyamides plasticized with H_2_O at different plasticization times; (**b**) and (**c**) are enlarged views of (**a**).

Figure 4 shows the FTIR spectra of BDIS (IA-80%) polyamides plasticized with three different plasticizers at different plasticization times. According to Hablot [28], the spectra of polyamides are characterized by six important absorption bands: the N–H stretching of amide at around 3300 cm^−1^, C=O stretching (amide II band) at around 1643 cm^−1^, N–H bending (amide II band) at around 1540 cm^−1^, C–C=O stretching at around 940 cm^−1^, and CH_2_ asymmetric deformation and symmetric deformation near 1461 and 1367 cm^−1^, respectively. With increasing plasticization time, the intensities of the characteristic absorption peaks of all plasticizers increase, indicating increasing content.

Figure 4a shows the FT-IR spectra of BDIS (IA-80%) polyamide plasticized with water. From Figure 4a we can see that with increasing plasticization time, the intensity and width of the peaks at around 3300 and 3500 cm^−1^ for the hydroxyl groups increase, indicating the formation of hydrogen bonds between the BDIS (IA-80%) polyamide and H_2_O molecules. According to Galdeano et al. [29], the absorption band for the O-H group in a hydrogen bond is between 3300 and 3600 cm^−1^. An intense broad band attributed to the complex vibrational stretching of free, inter- and intra-molecularly bound hydroxyl groups appears at about 3400 cm^−1^. The absorption band at 3409 cm^−1^ is characteristic of hydrogen-bonded –NH– groups or free H_2_O molecule. Unfortunately, the absorption bands at 3409 cm^−1^ overlap strongly and cannot be unambiguously resolved into their components. Generally, the amide groups of the polyamide easily form intramolecular hydrogen bonds while the introduction of H_2_O molecules favors the formation of intermolecular hydrogen bonds between the BDIS (IA-80%) polyamide and H_2_O molecules. It can be seen from Figure 4b,c that with increasing plasticization time, (i) a red shift occurs between the N–H stretching from 3311 to 3306 cm^−1^ and the C=O stretching (amide II band) from 1657 to 1632 cm^−1^; and (ii) the intensities of the asymmetric and the symmetric stretching vibration of aliphatic –CH_2_– (2853 and 2930 cm^−1^, respectively) decrease. These results indicate that water absorption increases the number of hydrogen bonds formed between the BDIS (IA-80%) polyamide and H_2_O molecules.

**Figure 5:**
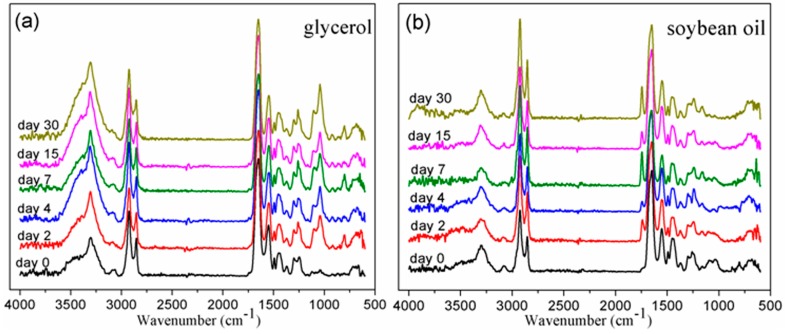
FTIR spectra of BDIS (IA-80%) polyamides plasticized with (**a**) glycerol and (**b**) soybean oil at different plasticization times.

Figure 5a shows the FTIR spectra of the BDIS (IA-80%) polyamide plasticized with glycerol. It can be seen from Figure 5a that with increasing plasticization time, the intensity of the –OH broad band at 3400 cm^−1^ increases. The intensity of the –CH_2_– band at 2930 and 2853 cm^−1^ and the peaks of the N–H stretching at 3311 cm^−1^ and C=O stretching (amide II band) at 1657cm^−1^ do not visibly change, an indication that only a small amount of hydrogen bonds between the BDIS (IA-80%) polyamide and the glycerol molecules are formed. Figure 5b shows the FTIR spectra of the BDIS (IA-80%) polyamide plasticized with soybean oil. It can be seen from Figure 5b that with increasing plasticization time, a new peak at 1747 cm^−1^, which is characteristic of the C=O stretching of the ester groups in soybean oil, but other absorption bands do not visibly change. Soybean oil does not cause large variations in the spectra, probably because the content of soybean oil is lowest of the three plasticizers used.

The three plasticizers have different interactions with the BDIS (IA-80%) polyamide. The plasticity of a plasticized polymer largely depends on the chemical composition, molecular weight, and functional groups of the plasticizer. The molecular weight and degree of branching of soybean oil are higher than those of glycerol and water; thus, it is difficult for the soybean oil molecules to penetrate the BDIS (IA-80%) polyamide molecular network. Additionally, the plasticizing effect of soybean oil is lower than that of glycerol because the content of soybean oil is lower than that of glycerol after plasticization for 30 days. Soybean oil has a low polarity, whereas water and glycerol are highly-polar molecules. BDIS polyamides are polar polymers, because of the existence of a large number of amide groups, which can interact more strongly with water and glycerol than soybean oil. That is, it is easy for a large number of small water molecules to occupy the intermolecular spaces between polymer chains, reducing the secondary forces between the chains and the energy required for molecular motion. As a result, the absorption of water transforms the BDIS (IA-80%) polyamide from a plastic to an elastomer.

**Figure 6:**
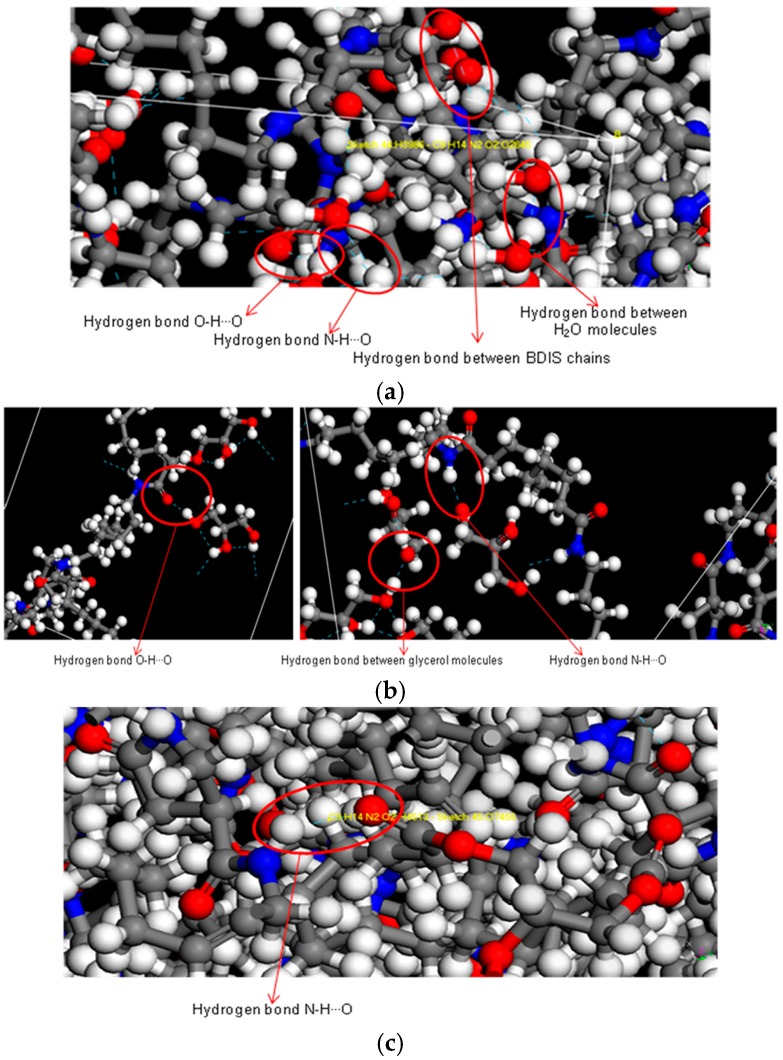
Formation of hydrogen bonds between BDIS (IA-80%) polyamide chains and plasticizer molecules as simulated by molecular modeling with Material Studio software (in the unit cell: red, O atom; white, H atom; gray, C atom; blue, N atom; the blue dashed lines represent hydrogen bonds): (**a**) Hydrogen bonds formed between BDIS (IA-80%) polyamide chains and H_2_O molecules; (**b**) hydrogen bonds formed between BDIS (IA-80%) polyamide chains and glycerol molecules; and (**c**) hydrogen bonds formed between BDIS (IA-80%) polyamide chains and soybean oil molecules.

The molecular dynamics simulation method was used to further study the formation of hydrogen bonds in the plasticized BDIS (IA-80%) polyamides. The BDIS (IA-80%) polyamide chains and plasticizer molecules were built in an amorphous cell to calculate the number, length, and angle of the hydrogen bonds. Figure 6 shows the formation of hydrogen bonds between BDIS (IA-80%) polyamide chains and plasticizer molecules after plasticization for 30 days. As shown in Figure 6a, three types of hydrogen bonds are formed between the BDIS (IA-80%) polyamide chains and H_2_O molecules: (І) the hydrogen bonds formed between the H_2_O molecules; (ІI) the hydrogen bonds N–H···O formed between the amino groups of the BDIS (IA-80%) polyamide chains and H_2_O molecules; and (ІІІ) the hydrogen bonds O···H–O between the carbanyl groups of the BDIS (IA-80%) polyamide chains and H_2_O molecules. It can be seen from Figure 6b that three types of hydrogen bonds are also formed between the BDIS (IA-80%) polyamide chains and glycerol molecules; however, most of the hydrogen bonds are formed between the glycerol molecules. From Figure 6c, we can see only one type of hydrogen bond, N–H···O, formed between the amino groups of BDIS (IA-80%) polyamide chains and soybean oil molecules. Due to the length and branched structure of soybean oil molecules, it is difficult for the soybean oil molecules to form hydrogen bonds with the BDIS (IA-80%) polyamide chains, and the number of hydrogen bonds, N–H···O, are significantly smaller than the number of hydrogen bonds formed between BDIS (IA-80%) polyamide chains and H_2_O or glycerol molecules. The number of hydrogen bonds follows the order H_2_O> glycerol> soybean oil, which is consistent with the results of FT-IR and plasticizer efficiency.

### 3.2. Solubility Parameters of BDIS (IA-80%) Polyamide and Plasticizers

Generally, polymer solubility can be estimated by using the solubility parameters calculated by the group contribution method. The interaction between the BDIS (IA-80%) polyamide and plasticizers were investigated by using the Hansen solubility parameter (HSP). The HSP [30] is one of the methods for analyzing the interaction between polymers and solvents. In a HSP analysis, all of the solvents have three parameters: the energy from dispersion bonds between molecules (δ_d_), the dipolar intermolecular force between molecules (δ_p_), and the hydrogen bonds between molecules (δ_h_). All of the solvents are characterized by a point in a three-dimensional structure, and δ_d_, δ_p_, and δ_h_ are plotted on three mutually-perpendicular axes. However, solubility parameters do not accurately reflect polymer solubility. The solubility of BDIS (IA-80%) polyamide was analyzed by using the HSP. The solubility parameters δ_d_, δ_p_, and δ_h_ of BDIS (IA-80%) polyamide in three different plasticizers are shown in Table 1. The HSP of BDIS (IA-80%) polyamide and three plasticizers have larger differences, indicating BDIS (IA-80%) polyamide is insoluble in the three plasticizers. The HSP of BDIS (IA-80%) polyamide was 28.9 (MJ/m^3^)^1/2^, which was calculated by the following equation:

(2) $$\delta_{t}^{2}=\delta_{d}^{2}+\delta_{p}^{2}+\delta_{h}^{2}$$

### 3.3. Thermal Properties of BDIS (IA-80%) Polyamide Plasticized with Different Plasticizers

**Figure 7:**
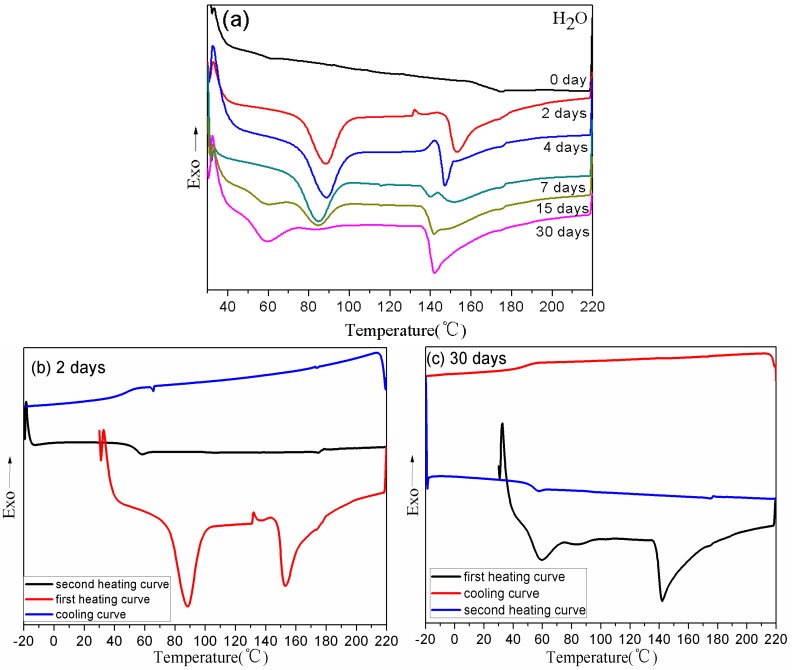
DSC curves of BDIS (IA-80%) polyamides plasticized with water: (**a**) first thermal scans of plasticized BDIS (IA-80%) polyamides at different plasticization times; (**b**) after being plasticized for two days, the heat-cool-heat cyclic experiments of BDIS (IA-80%) polyamides; and (**c**) after being plasticized 30 days, the heat-cool-heat cyclic experiments of BDIS (IA-80%) polyamides.

Figure 7 presents the DSC heating curves from the first thermal scans of the pure BDIS (IA-80%) polyamide and plasticized BDIS (IA-80%) polyamides at different plasticization times. The pure BDIS (IA-80%) polyamide shows a clear glass transition temperature (*T*_g_) at 59.0 °C and a low melting point at 175.0 °C. As shown in Figure 7a, two endothermic peaks appear at high plasticization times. The melting point of the plasticized BDIS (IA-80%) polyamide decreases from 175.0 to 141.5 °C. The endothermic peak, centered at about 88.0 °C is related to water evaporation. As shown in Figure 7b,c, the endothermic peak of water evaporating only appears in the first heating curves. Since the boiling point of water is 100 °C, and then if water-plasticized polymer is treated at higher temperature to remove the thermal history, water is also removed. Thus, in the second scan the effect of water plasticization disappears, and the data of first scan is discussed in the paper. From the second heating curves, BDIS (IA-80%) polyamides plasticized with water just show a clear glass transition temperature (*T*_g_) at around 57.0 °C and a low melting point at 175.0 °C as similar with the pure BDIS (IA-80%) polyamide. The enthalpy for this endothermic peak represents the energy required to vaporize the water present in the BDIS (IA-80%) polyamide. With increasing plasticization time, this endothermic peak of water evaporation decreases from 88.0 to 85.0 °C. After plasticization for 15 days, a small crystallization peak attributed to the water-induced crystallization of BDIS (IA-80%) polyamide appears at about 71.0 °C. The H_2_O molecules occupy the intermolecular spaces between the BDIS (IA-80%) polyamide molecular chains, so the amorphous molecular chains are activated into micro-Brownian motion. With the increase of temperature, the molecular chains realign, resulting in cold crystallization. Finally, the crystallization ends after the H_2_O molecules have evaporated. The different crystalline structures that can exist in BDIS (IA-80%) polyamide have been studied by XRD [31].

**Figure 8:**
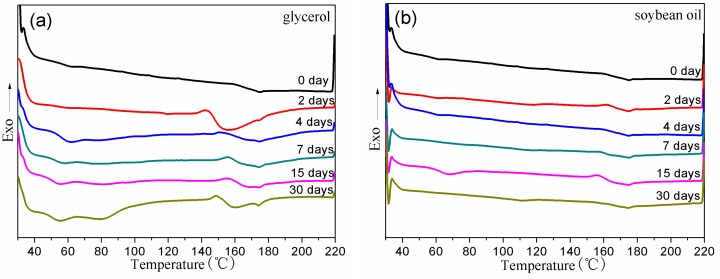
DSC curves of first thermal scans of BDIS (IA-80%) polyamides plasticized (**a**) glycerol and (**b**) soybean oil at different plasticization times.

The DSC curves of the BDIS (IA-80%) polyamide plasticized with glycerol are shown in Figure 8a. With increasing plasticization time, the glass transition temperature shifts from 60.0 to 55.2 °C. The endothermic peaks, centered at about 174.3, 159.2, and 80.5 °C, are related to the melting of BDIS (IA-80%) polyamide, the evaporation of glycerol, and the evaporation of water, respectively. The endothermic peak for water evaporation appears because the BDIS (IA-80%) polyamide and glycerol are prone to absorb water. Due to the hygroscopicity of BDIS (IA-80%) polyamide, the curves also show a cold crystallization peak at 65.6 °C after plasticization for seven days. It can be seen from Figure 8b that the curves for different plasticization times all have a similar appearance, displaying a glass transition temperature, the melting peaks of BDIS (IA-80%) polyamide, and no cold crystallization. Soybean oil plasticization does not change the DSC curve of BDIS (IA-80%) polyamide much, probably because the large molecular size and branched structure of soybean oil molecules make it difficult for the molecules to diffuse intothe polymer chains. Based on the thermal properties including the glass transition temperature, the efficiency of the plasticizer is in the order of H_2_O > glycerol > soybean oil, consistent with the FTIR results.

### 3.4. Thermal Stability

**Figure 9:**
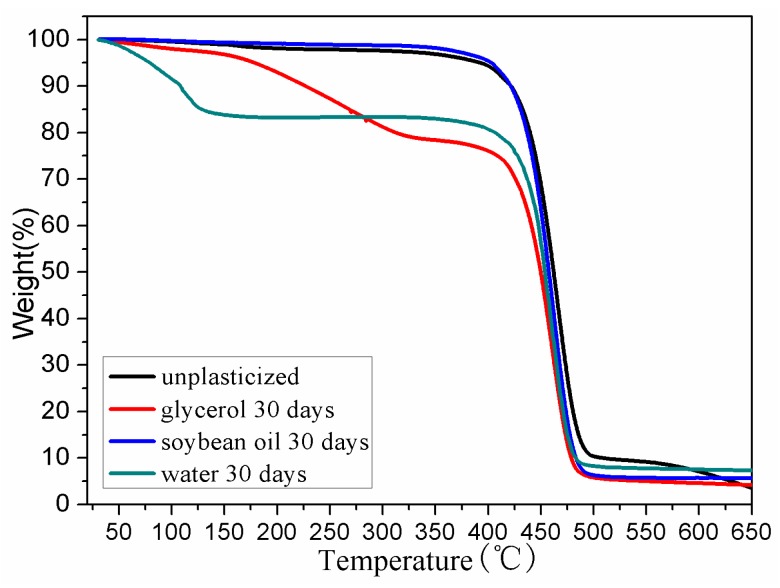
Thermogravimetric (TG) curves for unplasticized BDIS (IA-80%) polyamide and BDIS (IA-80%) polyamides plasticized with different plasticizers.

The thermal degradation temperatures of BDIS (IA-80%) polyamides plasticized with different plasticizers for 30 days were determined by thermogravimetric analysis (TGA) under a nitrogen atmosphere. Figure 9 shows the TGA curves of the plasticized BDIS (IA-80%) polyamides. There is only one degradation stage for the unplasticized BDIS (IA-80%) polyamide. The water- or glycerol-plasticized BDIS (IA-80%) polyamide presents two degradation stages. The first degradation stage from 50 to 148 °C for the water-plasticized BDIS (IA-80%) polyamide and that from 148 to 349 °C for the glycerol-plasticized BDIS (IA-80%) polyamide are ascribed to the volatilization of water and the volatilization or degradation of glycerol, respectively. TGA was also used to confirm the results from first thermal scans DSC measurements. The weight loss of the first degradation stage from 50 to 148 °C can be attributed to the evaporation of bound water and free water. This result is consistent with DSC first thermal scan measurement; the enthalpy for this endothermic peak of water evaporation with a shoulder beginning at 50 °C and centered at about 88.0 °C after plasticization for 30 days. The melting peak of water-plasticized polymer with a shoulder in the DSC measurements was also reported by Tanaka et al. [32,33]. Ping et al. [34] pointed out that the melting behavior of bound water in polymers can be attributed to the strong interactions of the water molecules with the polar groups of hydrophilic polymers. From the TGA curve we can see that the hydrated BDIS (IA-80%) polyamide shows an appreciable mass loss over 100 °C, and the mass loss is mainly ascribed to the volatilization of bound water in BDIS (IA-80%) polyamide. All of the degradation stages from 373–500 °C are the same for both BDIS (IA-80%) polyamides and attributed to the decomposition of BDIS (IA-80%) polyamide. The TGA curve of the soybean oil-plasticized BDIS (IA-80%) polyamide is similar to that of the unplasticized BDIS (IA-80%) polyamide. Thus, soybean oil-plasticized BDIS (IA-80%) polyamide has higher thermal stability than the other two plasticized BDIS (IA-80%) polyamides; it even has a slightly higher thermal stability than the unplasticized BDIS (IA-80%) polyamide. Below the BDIS (IA-80%) polyamide decomposition temperature, the mass loss of free water and crystallization water is 16.51%. The temperatures at 5% weight loss (*T*_5%_), maximum ratio of decomposition (*T*_dmax_), the temperatures at 95% weight loss (*T*_95%_), and residue mass are summarized in Table 2.

### 3.5. X-Ray Diffraction

**Figure 10:**
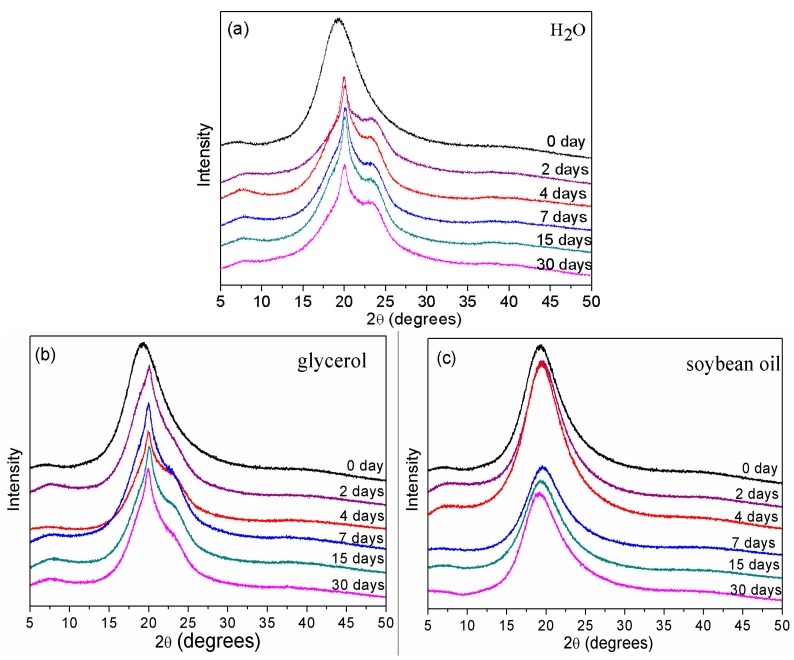
XRD patterns of BDIS (IA-80%) polyamides plasticized with three plasticizers at different plasticization times: (**a**) H_2_O; (**b**) glycerol; and (**c**) soybean oil.

The addition of plasticizer affects not only the thermal behavior but also the crystallization behavior of the BDIS (IA-80%) polyamide. To better understand the structure-property relationship of the BDIS (IA-80%) polyamides plasticized with three different plasticizers, we used XRD to study the crystallization behavior of the plasticized BDIS (IA-80%) polyamides. The XRD patterns of the BDIS (IA-80%) polyamides plasticized with three different plasticizers at different plasticization times are shown in Figure 10 for the 2θ range 5° to 50°. The patterns show a variety of crystal structures, mainly the α- and γ-crystalline phases and their variants. The α-crystalline phase consists predominantly of two characteristic diffraction peaks (the peaks at 20.2° and 23.9°), while the γ-crystalline phase consists predominantly of one characteristic diffraction peak (the shoulder at 21.3°) [35]. The XRD pattern for the unplasticized BDIS (IA-80%) polyamide presents a broad amorphous peak centered at 19.2°, mainly because the addition of IA increases molecular irregularity and reduces hydrogen bond interactions. The α-crystalline phase of the polyamides can be easily produced by solvent-induced crystallization or by slow crystallization from the melt [7]. The transition of the BDIS (IA-80%) polyamide from the amorphous to the water-induced crystalline state is clearly illustrated in Figure 10a. The two diffraction peaks at 20.0° and 23.5° can be assigned to the α-crystalline phase for the plasticized BDIS (IA-80%) polyamides. With increasing plasticization time, the intensity of the diffraction peaks first slightly increases, and then remains basically unchanged after plasticization for four days. The transition of the amorphous form to the α-crystalline phase also indicates that the H_2_O molecules improve the crystallizability of BDIS (IA-80%) polyamide, consistent with the DSC results. Since the H_2_O molecules enter the amorphous region in the BDIS (IA-80%) polyamide, the intermolecular hydrogen bonds are replaced by interactions of the guest H_2_O molecule bound to the BDIS (IA-80%) polyamide. The interaction of an amide group with a H_2_O molecule is significantly stronger than the hydrogen bond between CO and NH; thus, the highly anisotropic BDIS (IA-80%) polyamide chains are realigned.

The XRD patterns of the BDIS (IA-80%) polyamide plasticized with glycerol are shown in Figure 10b. We can see that the effects of glycerol on the crystallization of BDIS (IA-80%) polyamide are similar to those of H_2_O. With increasing plasticization time, first the broad diffraction peak transforms into a sharp peak, and then a weak shoulder peak appears on the right side of the sharp peak. This behavior is probably due to the hygroscopicity of both BDIS (IA-80%) polyamide and glycerol, which are saturated with water to achieve equilibrium with the environment. This behavior also means that H_2_O molecules induced BDIS (IA-80%) polyamide crystallization. As shown in Figure 10c, all of the XRD patterns of BDIS (IA-80%) polyamide plasticized with soybean oil display a broad amorphous peak centered at 19.2°. Due to the large size of its molecules and its high degree of branching, soybean oil cannot easily diffuse through the BDIS (IA-80%) polyamide molecular chains. Therefore, soybean oil is just a plasticizer, but cannot induce BDIS (IA-80%) polyamide crystallization. The transition of the amorphous form to the α-crystalline phase also indicates that the efficiency of the plasticizer for BDIS (IA-80%) polyamide decreases with the increasing size of the plasticizer molecule, consistent with the DSC results.

### 3.6. Polarized Optical Microscopy

**Figure 11:**
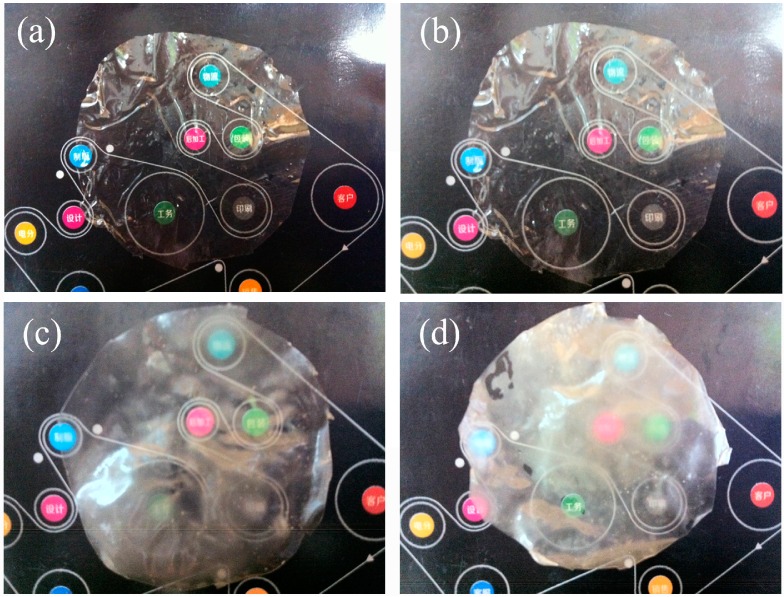
Photos of water-induced crystallization of BDIS (IA-80%) polyamide: (**a**) 0 h; (**b**) 24 h; (**c**) 48 h; and (**d**) 72 h.

POM was used to study the H_2_O-induced crystallization and the structure-property relationship of the H_2_O-plasticized BDIS (IA-80%) polyamide. Figure 11 shows the photos of the water-induced crystallization of BDIS (IA-80%) polyamide. The unplasticized BDIS (IA-80%) polyamide films are clear and transparent. The BDIS (IA-80%) polyamide films were immersed in water for water-induced crystallization testing. After an immersion time of 72 h, the water-induced crystallization of BDIS (IA-80%) polyamide has reached equilibrium. The transition of the transparent films to creamy white films indicates that the water-plasticized BDIS (IA-80%) polyamide transformed from an amorphous material to a crystalline material. The crystallizability of BDIS (IA-80%) polyamide increases with increasing H_2_O content. The unplasticized BDIS (IA-80%) polyamide films have an amorphous structure and no crystalline domains are observed. By contrast, the films with water-induced crystallization have covered the background various colored circles similar to those reported by other researchers [36,37].

**Figure 12:**
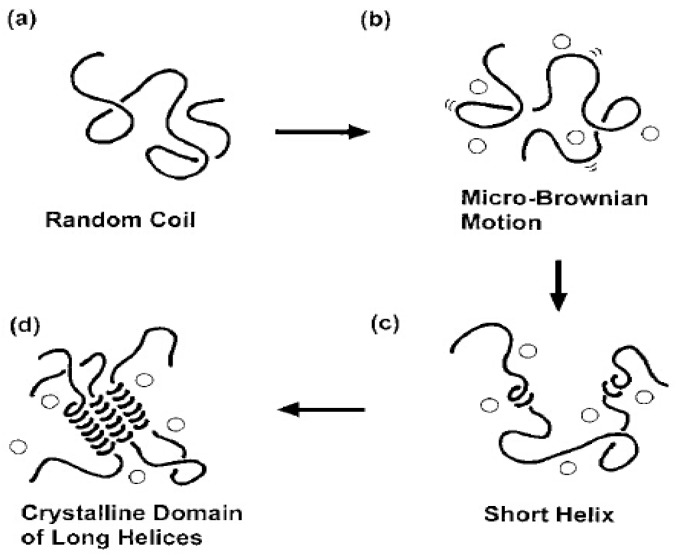
Schematic illustration of structural change in the process of water-induced crystallization of amorphous BDIS (IA-80%) polyamide. (**a**) Random coil; (**b**) Micro-Brownian motion; (**c**) Short helix; (**d**) Crystalline domain of long helices.

After the addition of water, the mobility of the BDIS (IA-80%) polyamide chains increases even below *T*_g_ of the polyamide. The random coils in the amorphous region are activated into micro-Brownian motion by absorbing water molecules, and then short, regular, helical segments are generated [36,38]. These short helices function as nuclei on which longer helical segments are grown, and a crystalline lattice is formed, as seen in Figure 12. The H_2_O-induced crystallization of BDIS (IA-80%) polyamide leads to microcrystals rather than full spherulites below *T*_g_.

### 3.7. Mechanical Properties

**Figure 13:**
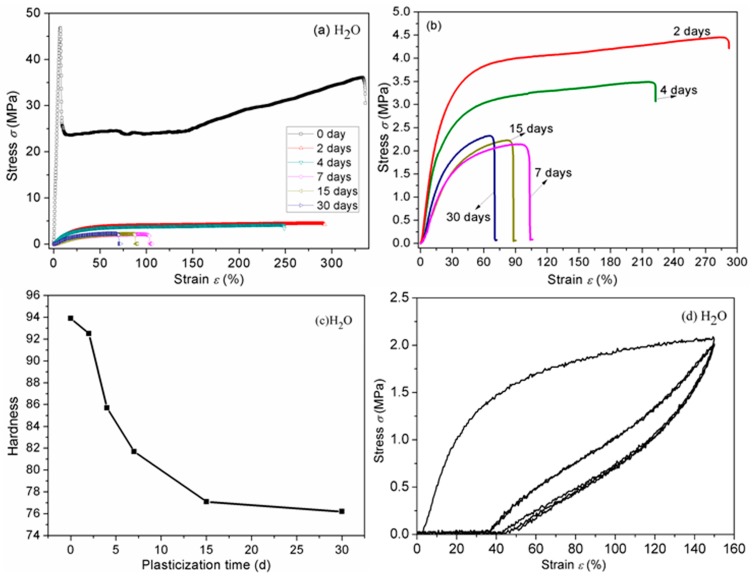
(**a**) Stress-strain curves for BDIS (IA-80%) polyamides plasticized with H_2_O at different plasticization times; (**b**) enlarged view of (**a**); (**c**) hardness curve for BDIS (IA-80%) polyamides plasticized with H_2_O at different plasticization times; and (**d**) cyclic loading-unloading curves for BDIS (IA-80%) polyamide soaked in water for two days.

Figure 13 shows the stress-strain curves and hardness of the BDIS (IA-80%) polyamides with water at different plasticization times. The BDIS (IA-80%) polyamides exhibit excellent mechanical properties, including high modulus, yield stress, tensile strength, and elongation at break. It can be seen from Figure 13a that for the water-plasticized BDIS (IA-80%) polyamide, the yield point disappears and the Young’s modulus and the elongation at break decrease significantly after two days of H_2_O absorption. Hydration can partially destroy the hydrogen bonds of BDIS (IA-80%) polyamides. The weakening of the hydrogen bonds increases chain mobility, swells the polymer, decreases the *T*_g_ of the polyamide, and transforms the material from a plastic to an elastomer. With the plasticization time increasing, the Young’s modulus and the elongation at break of water-plasticized BDIS (IA-80%) polyamide further decrease.

After 30 days of H_2_O absorption, the elongation at break decreases from 350% to 70% (see Figure 13b) and the hardness decreases from 93.9 to 76.2 (see Figure 13c). The pure BDIS (IA-80%) polyamides are linear amorphous toughness materials. After the material was plasticized in water for two days, the water absorption reached 13%. Compared with the pure BDIS polymer, in the stress-strain relationship cure of the sample after two days water absorption, the yielding of a plastic material disappeared, the modulus decreased significantly, and the elongation at break was still as high as, or close to, 300%, which was the transition from plastic to soft elastomer of plasticized BDIS (IA-80%) polyamide. According to theory of plasticization [39,40], the plasticizer is absorbed by matrices when blending with the polymer, which can dissolve the connection points of the polymer macromolecule. As the connection points are destroyed, the centers of force that make the polymer macromolecules move closer together are masked, leading to a weakening of the attractive force between polymer macromolecules, and tensile strength of polymer decreases. Usually, any plasticizer can weaken the mechanical properties of polymers when too much is added. As the water absorption time was extended, the uptake of water was further increased, and the interaction between macromolecules of BDIS (IA-80%) polyamide was further weakened by water swelling and plasticization. As the swelling ratio of water-plasticized BDIS (IA-80%) polyamide further increased, the spacing between polymer molecular chains were further increased. The H_2_O molecules, as a plasticizer, gradually became an impurity that deteriorates the BDIS (IA-80%) polyamides. Since BDIS (IA-80%) polyamides is not crosslinked, the mechanical properties decreased, as well as the elongation. On one hand, the increase in crystallinity as indicated by the DSC and XRD results should be one reason for the increase of the modulus. On the other hand, the H_2_O molecule act as a plasticizer to facilitate chain slip under the lubrication effect and disrupt H_2_O-induced crystallization. The cyclic loading-unloading stress-strain curves (Figure 13d) of the H_2_O-plasticized samples show the typical characteristics of a rubbery material, different from the yield behavior observed for the hydrated samples. The hydrated BDIS (IA-80%) polyamide can be easily twisted into various shapes. Since the BDIS (IA-80%) polyamides are totally amorphous, their H_2_O absorption is very high. The common moisture uptake of polyamides ranges from 1.2% to 3.0% [4]. The moisture uptake of unplasticized BDIS (IA-80%) polyamide after 30 days reaches an unusually high value of almost 25.0%, higher than those of traditional polyamides, such as PA 66 and PA 6. Hydrogen bonds still exist between the H_2_O molecules and the macromolecules, so the rubbery material has a high elasticity, even though no chemical crosslink exists. Rare among polyamides, this H_2_O-plasticized BDIS (IA-80%) polyamide may be useful for the preparation of a physical water gel.

**Figure 14:**
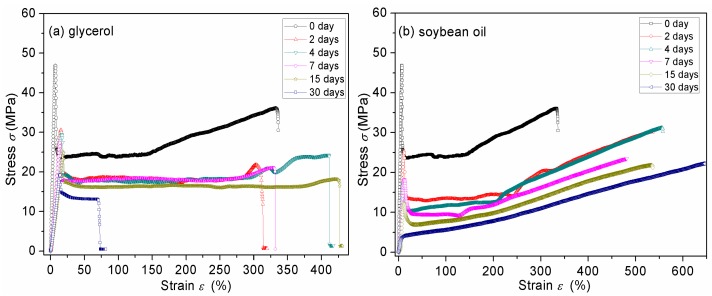
Stress-strain curves for BDIS (IA-80%) polyamides plasticized with glycerol (**a**) and soybean oil (**b**) at different plasticization times.

From Figure 14a,b we can see that the samples plasticized with glycerol or soybean oil show a yield behavior and high elongation at break as the unplasticized samples. Generally, with increasing plasticization time, the elongation at break and tensile toughness of BDIS (IA-80%) polyamides increase, but the Young’s modulus and ultimate tensile strength decrease. The difference between the elongation at break is that after 15 days of plasticization, the elongation at break of the BDIS (IA-80%) polyamide plasticized with glycerol starts to decrease, but the elongation at break of the BDIS (IA-80%) polyamide plasticized with soybean oil keeps on increasing. After 30 days of soybean oil absorption, the plasticized BDIS (IA-80%) polyamide displays excellent toughness and an elongation at break of 650%. The Young’s modulus and yield point decrease significantly as the plasticizer concentration increases for both the glycerol- and the soybean-oil-plasticized samples. The high elongation at break can be explained by the orientation of the macromolecular chains along the stretching direction under external stresses and the lubrication effect of the plasticizer molecules.

Plasticizer content is an important estimate of the plasticizer efficiency. The plasticizer content of BDIS (IA-80%) polyamide related to the plasticization time is shown in Table 3. With increasing plasticization time, the content of the three plasticizers increase. At the same plasticization time, the plasticizer content of BDIS (IA-80%) polyamide plasticized with soybean oil is lower than that of the BDIS (IA-80%) polyamide plasticized with water or glycerol. The BDIS (IA-80%) polyamide has a water uptake of almost 25.0% after being soaked in water for 30 days.

## 4. Conclusions

In the present work, the efficiencies of the plasticizer for BDIS (IA-80%) polyamide follow the order of H_2_O > glycerol > soybean oil. The addition of a plasticizer resulted in plasticization of the polymer matrix, as reflected by the lower T_g_ and tensile strength, and elongation at break. The BDIS (IA-80%) polyamides showed considerable water absorption, up to 25.0%. The H_2_O-plasticized BDIS (IA-80%) polyamides showed rubber-like properties, rarely reported for polyamides. These plasticized amorphous BDIS (IA-80%) polyamides, which were obtained from biomass monomers, exhibit versatility in physical characteristics and mechanical properties; thus, they could find wide applications. Although the BDIS (IA-80%) materials are linear materials, the water molecules will evaporate, but they could still be used in applications in an aqueous environment which does not need to consider the evaporation of water, such as contact lenses and water filtration membrane material. In addition, the water-plasticized amorphous BDIS (IA-80%) polyamides showed good bio-compatibility, which makes them useful for applications in biomedical materials. The soybean oil-plasticized BDIS (IA-80%) polyamides displayed excellent toughness, with elongation at break values reaching 650%. Additionally, soybean oil-plasticized BDIS (IA-80%) polyamide had higher thermal stability than the H_2_O-plasticized BDIS (IA-80%) polyamide or glycerol-plasticized BDIS (IA-80%) polyamide, and even the unplasticized BDIS (IA-80%) polyamide.

## Figures and Tables

**Table 1:** Hansen solubility parameter of the three plasticizers.

Plasticizers	δ_d_ (MJ/m^3^)^1/2^	δ_p_ (MJ/m^3^)^1/2^	δ_h_ (MJ/m^3^)^1/2^	δ_t_ (MJ/m^3^)^1/2^
Water	15.5	16.0	42.4	47.9
Glycerol	17.3	12.1	29.3	43.2
Soybean oil	17.1	0.5	2.8	17.3

**Table 2:** Main degradation temperatures and residue mass for BDIS (IA-80%) polyamides with different plasticizers.

Samples	*T*_5%_ (°C)	*T*_dmax_ (°C)	*T*_95%_ (°C)	Residue mass (%)
BDIS (IA-80%)	403	459	650	5.58
Water 30 days	79	458	601	7.28
Glycerol 30 days	179	460	538	4.14
Soybean oil 30 days	391	466	630	3.52

**Table 3:** Plasticizer contents of BDIS (IA-80%) polyamide at different plasticization times.

Plasticizers	2days	4days	7days	15days	30days
Water	11.5 ± 0.8	15.0 ± 0.7	18.5 ± 0.7	20.9 ± 0.6	25.0 ± 0.6
Glycerol	4.0 ± 0.5	5.3 ± 0.6	6.6 ± 0.4	8.7 ± 0.4	9.4 ± 0.5
Soybean oil	2.3 ± 0.4	4.0 ± 0.4	5.2 ± 0.7	6.0 ± 0.5	6.9 ± 0.5

## Supplementary Materials

The following are available online at www.mdpi.com/2073-4360/8/7/257/s1, Figure S1: Optical micrographs of water plasticized BDIS (IA-80 %) after 30 days in vitro cytotoxicity assay (L929 imaged after 72 h of culture with test materials).
