# Supplementary material for: Preparation of Bio-Based Polyamide Elastomer by Using Green Plasticizers
Source: Polymers (Basel). 2016 Jul 14;8(7):257. doi: 10.3390/polym8070257 (PMC6432398; doi:10.3390/polym8070257)
Supplement: Supplementary file 1 [file polymers-08-00257-s001.pdf]

Supplementary Materials: Preparation of Bio-Based Polyamide Elastomer by Using Green Plasticizers

Miaomiao He, Zhao Wang, Runguo Wang, Liqun Zhang and Qingxiu Jia

This work studied plasticized BDIS polymer which was obtained from biomass monomers and water plasticized BDIS showed good bio-compatibility(as shown in Figure S1), which makes it useful for applications such as in contact lenses and biomedical materials (carrier materials of externally applied medicine and even tissue engineering materials).

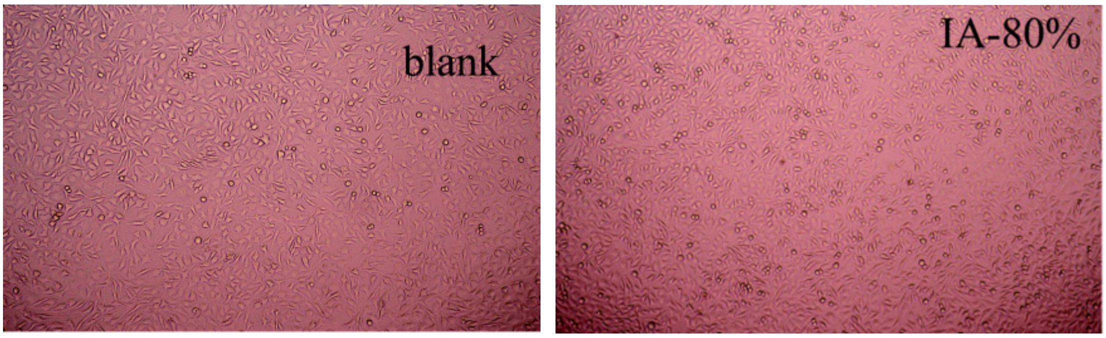

Figure S1. Optical micrographs of water plasticized BDIS (IA-80 %) after 30 days in vitro cytotoxicity assay (L929 imaged after 72 h of culture with test materials).
